# Diabetes and the Developing Mind: Intelligence Quotient (IQ) and Its Associations in Indian Children and Youth With Type 1 Diabetes

**DOI:** 10.7759/cureus.90438

**Published:** 2025-08-18

**Authors:** Sonali S Wagle-Patki, Amarpaul Keerthi, Aboli A Bhalerao, Monika Sawale, Vaman V Khadilkar, Anuradha V Khadilkar, Rujul R Potnis

**Affiliations:** 1 Department of Growth and Pediatric Endocrinology, Hirabai Cowasji Jehangir Medical Research Institute, Pune, IND; 2 Department of Health Sciences, Savitribai Phule Pune University, Pune, IND

**Keywords:** adolescents, aiq, binet-kamat test, children, glycemic control, india, intelligence quotient, type 1 diabetes, youth

## Abstract

Background

Children’s verbal intelligence quotient (IQ) may be affected by type 1 diabetes (T1D), potentially leading to a decrease in IQ. Studies have reported associations between IQ and both the age at onset of T1D and glycemic control. However, there are very few studies on children and youth with T1D from India. Early diagnosis of cognitive impairment would allow for timely intervention. Therefore, we aimed to study IQ and its predictors in children and youth with T1D.

Methods

In this clinic-based cohort study, sociodemographic/educational, anthropometric, lifestyle, and glycemic control details were recorded. The IQ of 413 participants (aged 4-22 years) was assessed using the standardized Binet Kamat Test. Adjusted IQ (AIQ) was computed using age-appropriate formulae. Associations between AIQ and its determinants/predictors were examined using correlation analysis, followed by linear regression analysis, adjusting for possible confounders.

Results

Study participants (median age: 14.0 years (IQR: 11.0-17.2)) demonstrated average cognitive performance based on standardized AIQ categories, with a median AIQ of 102 (IQR: 91-117). Higher AIQ scores were associated with younger age (standardized β = -0.207) and higher maternal (β = 0.153) and paternal education (β = 0.100). Conversely, poorer glycemic control (glycosylated hemoglobin (HbA1c); β = -0.133) and longer duration of diabetes (β = -0.154) were associated with lower AIQ. In multiple regression analysis, age, duration of diabetes, glycemic control, and maternal education emerged as independent determinants of AIQ in children, adolescents, and youth with T1D.

Conclusions

Participants with T1D had an average IQ. Glycemic control, age, duration of diabetes, and parental education were important predictors of IQ. Our findings highlight the need for screening for cognitive impairment and emphasize the importance of good glycemic control to optimize IQ in individuals with T1D.

## Introduction

Type 1 diabetes (T1D) is a chronic autoimmune condition resulting from the destruction of insulin-producing β-cells in the pancreas. It often manifests during childhood or adolescence and requires lifelong insulin therapy. According to the International Diabetes Federation, India has the highest number of children living with T1D, with an estimated average prevalence of 24.0 cases per 1,000 children per year [1]. In addition to metabolic and physical health challenges, T1D is increasingly recognized for its potential impact on neurocognitive development in children and adolescents. Several studies suggest that glycemic dysregulation, including chronic hypoglycemia, recurrent hypoglycemia, and glycemic variability, during sensitive periods of brain maturation may have detrimental effects on cognitive function in children with T1D [2,3].

Over the past few decades, international research has consistently shown that children and adolescents with T1D may experience mild yet meaningful difficulties in specific areas of cognitive functioning. Meta-analytic findings indicate that children with T1D tend to perform slightly lower than their peers in domains such as attention, executive function, and memory, particularly when diabetes onset occurs at a younger age [4]. Similarly, a systematic review of 33 studies found deficits in processing speed, psychomotor efficiency, and cognitive flexibility in youth with T1D, with these effects more closely associated with microvascular complications than with severe hypoglycemic episodes [5]. While general intelligence and memory are often preserved, a cohort of young adults with early-onset T1D showed poorer performance on tasks requiring fluid intelligence and executive control, despite having average full-scale intelligence quotient (IQ) scores [6]. Together, these findings suggest that subtle impairments, especially in higher-order cognitive skills, may emerge in children with T1D as a result of long-term glycemic dysregulation during critical periods of brain development.

In the Indian context, efforts to assess such cognitive differences among the general population have relied heavily on culturally adapted tools, with the Binet Kamat Test (BKT) of Intelligence being one of the most widely used instruments. Adapted from the Stanford-Binet scale, the BKT has remained a staple in clinical and research settings due to its affordability, ease of administration, and relevance for children across diverse linguistic and socioeconomic backgrounds [7]. Despite being standardized in the 1960s, the BKT continues to be a valid tool for cognitive assessment, particularly in low-resource environments. Nevertheless, the lack of a formal revision or re-standardization remains a significant limitation. To address this, certain methodological adjustments are needed when interpreting BKT scores today. The test uses a standard deviation of 18.7, differing from the 15-point standard recommended by WHO and ICD-10 guidelines. To improve comparability with contemporary scales, prorated IQ scores are often calculated. Additionally, the Flynn effect, which describes the trend of rising IQ scores over generations, suggests that results based on the BKT’s outdated norms may underestimate current cognitive ability by approximately 15 points. To mitigate this effect, a formula has been proposed to adjust IQ scores and compensate for outdated normative data [8]. While such adjustments require careful interpretation, especially in populations from lower socioeconomic strata, the BKT remains a contextually appropriate and widely recognized tool for evaluating intelligence in Indian children. Its applicability was reaffirmed in a recent study involving preschool-aged children from rural Konkan, Maharashtra, where it was used to evaluate IQ despite a high prevalence of malnutrition. Interestingly, the study found no significant relationship between IQ scores and anthropometric measures, birth weight, or dietary intake, supporting the test’s robustness in evaluating cognitive functioning independently of nutritional status [9].

To bridge these gaps, the present study examined adjusted IQ (AIQ) in a clinic-based cohort of children and youth with T1D in Western India, in line with Roopesh and Kumble’s (2016) recommendation [8]. Many participants belonged to lower socioeconomic groups, providing a more inclusive representation. This study aimed to explore the association between glycemic control, sociodemographic variables (such as parental education), and cognitive outcomes in this cohort. To the best of our knowledge, this is the first Indian study to evaluate cognitive functioning in children with T1D using the BKT with such adjustments. By doing so, it contributes to the limited but growing literature on the neurocognitive impact of T1D in Indian pediatric populations and situates these findings within both national and global contexts.

## Materials and methods

This was a clinic-based cohort study conducted at a charity clinic for children with diabetes at a tertiary care center in Western Maharashtra. The “Sweetlings” program is designed to provide holistic care for children with T1D, aiming to support underprivileged children and youth (up to 22 years) with the condition. The study was conducted between September 2022 and April 2023.

Children and youth aged 4-22 years were eligible to participate. Exclusion criteria included being outside the target age range, having a diagnosed intellectual disability, declining assent, or lack of parental/guardian consent. All children with T1D enrolled in the Sweetlings program for the 2022-2023 annual check-up (n = 510) were invited to participate. Of these, children younger than five years (n = 60) and older than 22 years (n = 20) were excluded. From the remaining 430 children, 17 could not attend the annual check-up due to logistical reasons. Thus, IQ was assessed in 413 participants (4-22 years) using the standardized BKT. AIQ was computed using age-appropriate formulae.

Ethical approval was obtained from the Institutional Biomedical and Health Research Ethics Committee (JCDC/BHR/23/025). Written informed consent was obtained from a parent or guardian, and written informed assent was obtained from children aged over 10 years.

Participants were examined for diabetes-related complications, body size and composition, and cognitive function, primarily IQ. Because cognitive testing was a key component of the study, assessments were conducted in a separate, quiet room to ensure a conducive environment. Sociodemographic information (age, sex, parental education, and occupation) was collected using a standard questionnaire. Anthropometric measurements included height, weight, waist circumference, and hip circumference. Height was measured using a Seca portable stadiometer (accuracy up to 0.1 cm) and body weight using a Seca 876 flat scale (accuracy up to 100 g). BMI was calculated as weight (kg /height (m²).

Glycemic control was assessed by measuring glycosylated hemoglobin (HbA1c) using high-performance liquid chromatography (Bio-Rad Laboratories GmbH, Feldkirchen, Germany). Lifestyle factors, including physical activity levels and sleep duration, were collected using standardized questionnaires [10].

IQ was assessed using the BKT of Intelligence (version 4), purchased from Prasad Psycho Corporation and used under a permitted research license. The full test items are not reproduced due to copyright restrictions. The BKT is an Indian adaptation of the Stanford-Binet scale, modified to measure the intelligence of Indian children. It is an age-scale test, with items grouped into age levels ranging from three years to a superior adult level. Each age level contains six subtests, assessing both verbal and performance abilities. The BKT estimates mental age and IQ for individuals aged 3-22 years.

The test evaluates six main cognitive factors: language, memory, conceptual thinking, reasoning, visual-motor skills, and social intelligence, and five subcognitive factors: meaningful memory, non-meaningful memory, non-verbal reasoning, verbal reasoning, and numerical reasoning. Reported reliability exceeds 0.7, and validity compared with teacher-estimated IQ in healthy children is 0.5 [9].

**Figure 1:**
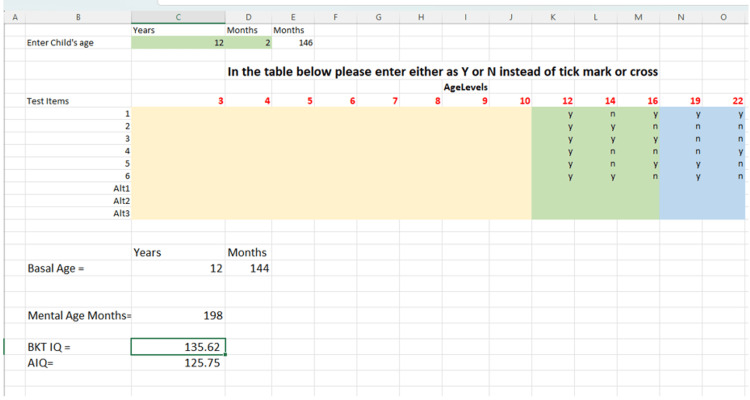
Calculations using the macro developed in Excel The figure shows the details of the calculations performed using a macro developed in an Excel sheet.

Using age-specific formulae described by Roopesh and Kumble [8], we developed an Excel macro (Microsoft Corporation, Redmond, WA, USA) to calculate AIQ (Figure 1).

Furthermore, the Stanford-Binet Fifth Edition classification was used for analysis [12]. Data are presented as median (25th and 75th percentile) values and as percentages for frequencies. The chi-square test was used to compare categorical outcomes between groups, and medians were compared using the nonparametric Mann-Whitney U test. Statistical analysis was performed using IBM SPSS Statistics for Windows, Version 25.0 (Released 2017; IBM Corp., Armonk, NY, USA). Given an alpha level of 5% and assuming a small effect size of 0.12, a sample size of 425 was sufficient to achieve a power of 0.8.

Correlations between demographic factors, lifestyle factors, HbA1c, and anthropometric parameters with IQ scores were calculated using Spearman’s correlation coefficient. Predictors of IQ were assessed using multiple linear regression, with exposures including the child’s age, parental education, HbA1c, duration of diabetes, and lifestyle factors. The outcome variable was IQ score.

## Results

**Figure 2:**
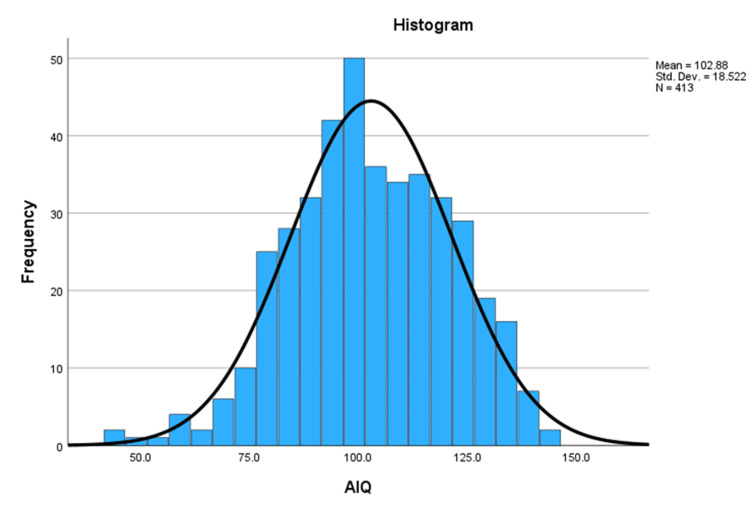
Distribution of AIQ scores in children and youth with T1D This histogram illustrates the distribution of AIQ scores in the study population, showing an approximately normal distribution centered around the mean AIQ. AIQ, adjusted intelligence quotient; T1D, type 1 diabetes

A total of 413 children and adolescents with T1D (median age, 14.0 years; IQR: 11.0-17.2; 190 males, 223 females) were assessed using prorated or AIQ scores. The overall median AIQ was 102 (IQR: 91-117), indicating cognitive performance within the normal range for most participants. Only a small subset achieved scores above 129 (Figure 2).

No significant differences were found between males and females in AIQ scores or in demographic, anthropometric, or biochemical characteristics (Table 1).

**Table 1:** Demography, anthropometry, and biochemical characteristics of children with T1D Values are presented as median (25th-75th percentile). The nonparametric Mann-Whitney U test was used to compare medians and calculate p-values. A z-test for proportions was used to compare pubertal stage-wise percentages between males and females. Scores were derived using the BKT of Intelligence, ^©^ Prasad Psycho Corporation, 2019, New Delhi. Used with permission for academic research. Questionnaire items are not reproduced due to copyright restrictions. AIQ, adjusted intelligence quotient; BKT, Binet Kamat Test; HbA1c, glycated hemoglobin; SES, socioeconomic status; T1D, type 1 diabetes; WHR, waist-to-hip ratio

Parameter	Male (n = 190)	Female (n = 223)	Total (n = 413)	Z value	p
Age (years)	14.7 (11.1-17.2)	13.6 (10.9-17.1)	14.0 (11.0-17.2)	-0.92	0.358
Education of father (years)	10.0 (8.0-12.0)	10.0 (10.0-12.0)	10.0 (8.0-12.0)	-2.055	0.04
Education of mother (years)	10.0 (8.0-12.0)	10.0 (8.0-12.0)	10.0 (8.0-12.0)	-1.691	0.091
SES score	10.0 (9.0-12.0)	10.0 (8.3-11.0)	10.0 (9.0-11.0)	-0.675	0.5
Pubertal stage					
Stage 1	34 (17.9%)	47 (21.1%)	84 (19.6%)	-2.007	0.044
Stage 2	34 (17.9%)	30 (13.5%)	64 (15.5%)	0.707	0.479
Stage 3	23 (12.1%)	18 (8.1%)	41 (9.9%)	1.104	0.269
Stage 4	24 (12.6%)	9 (4.0%)	33 (8.0%)	3.692	<0.001
Stage 5	75 (39.5%)	119 (53.4%)	194 (47.0%)	-4.467	<0.001
Duration of diabetes (years)	5.6 (2.6-8.4)	6.2 (3.1-9.0)	5.8 (3.1-8.8)	-0.626	0.531
Height z-score	-0.78 (-1.34, -0.19)	-0.70 (-1.34, -0.04)	-0.73 (-1.34, -0.11)	-0.88	0.379
Weight z-score	-0.68 (-1.39, 0.08)	-0.74 (-1.34, 0.13)	-0.74 (-1.36, 0.11)	-0.357	0.721
BMI z-score	-0.66 (-1.17, -0.05)	-0.39 (-1.02, 0.15)	-0.57 (-1.10, 0.06)	-2.186	0.029
Waist circumference z-score	-1.25 (-1.82, -0.50)	-1.03 (-1.63, -0.39)	-1.15 (-1.78, -0.41)	-1.62	0.105
WHR	0.84 (0.80-0.87)	0.83 (0.79-0.87)	0.84 (0.83, 0.86)	-0.614	0.539
AIQ	106.0 (93.0-117.3)	101.0 (89.0, 116.0)	102.0 (91.0-117.0)	-1.565	0.118
HbA1c (%)	9.7 (8.5-11.2)	9.5 (8.4-11.2)	9.6 (8.5-11.2)	-0.814	0.416
Total screen time (min/day)	120.0 (90.0-188.6)	120.0 (64.3-180.0)	120.0 (77.1-186.0)	-1.473	0.141
Total sleep time (min/day)	531.4 (480.0-565.7)	540.0 (485.7-557.1)	540.0 (480.0-557.1)	-0.571	0.568
Total moderate-to-vigorous activity (min/day)	51.4 (30.0-85.7)	32.1 (17.1-51.4)	40.0 (21.4-64.3)	-5.614	<0.001

**Figure 3:**
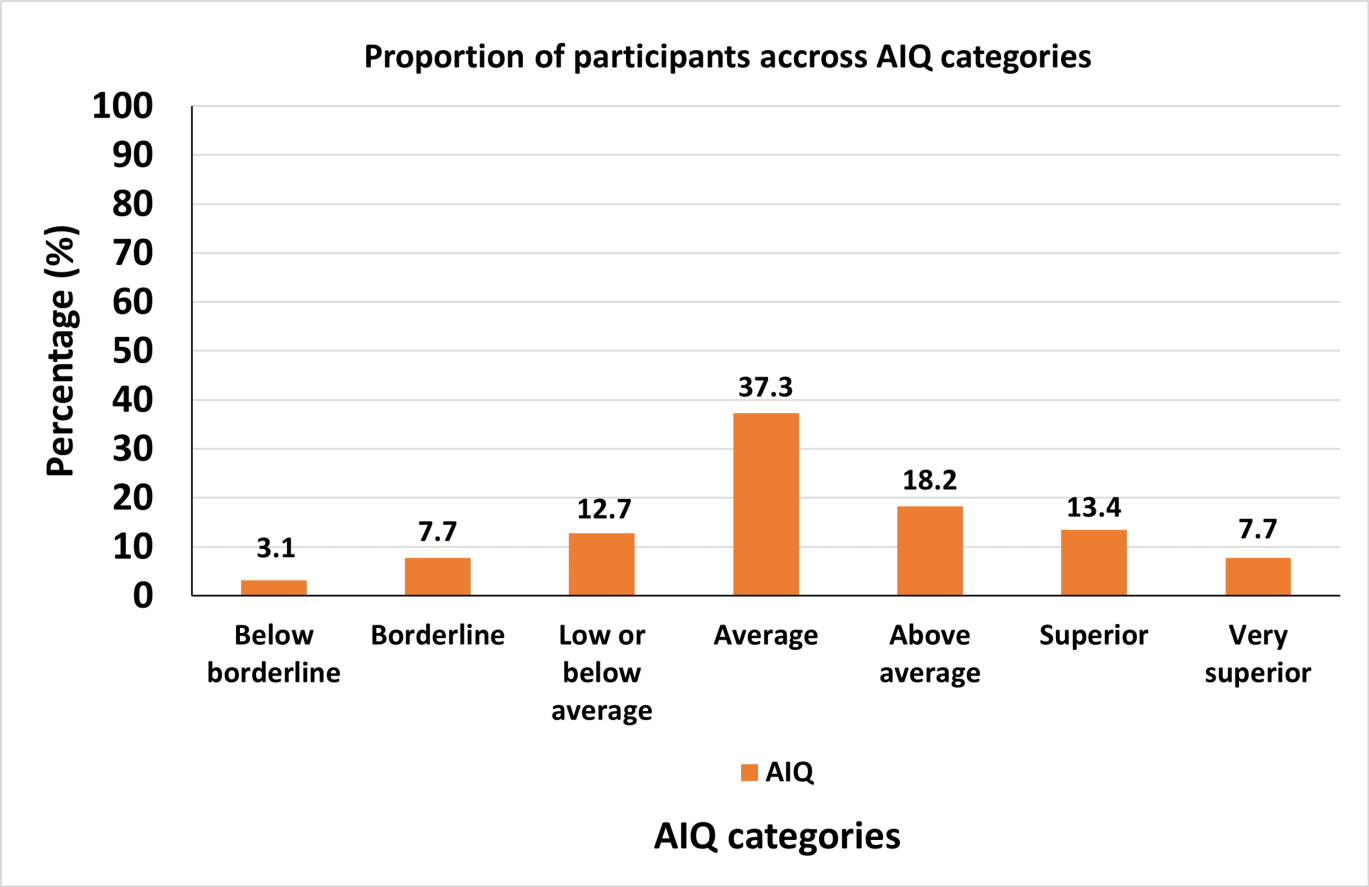
Distribution of children and youth with T1D across AIQ categories Bar chart showing the percentage of participants classified into standardized AIQ categories (e.g., below average, average, and above average), reflecting cognitive diversity within the cohort. AIQ, adjusted intelligence quotient; T1D, type 1 diabetes

Approximately 38% of participants fell within the defined average AIQ category (Figure 3).

**Figure 4:**
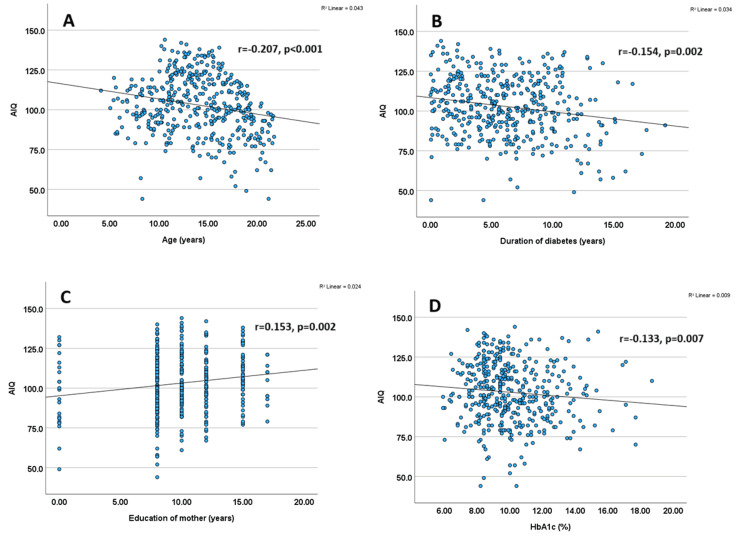
Correlation of demographic factors, HbA1c, and duration of diabetes with AIQ scores (A) Correlation between AIQ and age: Scatter plot illustrating the association between chronological age and AIQ scores in children and youth with T1D. The trend line indicates the direction and strength of the correlation. (B) Correlation between AIQ and duration of diabetes: Scatter plot showing the relationship between diabetes duration and AIQ scores, assessing potential cognitive effects of long-term disease exposure. (C) Correlation between AIQ and maternal education: Scatter plot demonstrating the association between years of maternal education and AIQ scores in children with T1D, highlighting the potential role of maternal education in cognitive outcomes. (D) Correlation between AIQ and HbA1c: Scatter plot showing the relationship between glycemic control (HbA1c levels) and AIQ scores in children with T1D, with the trend line suggesting the nature of the association. AIQ, adjusted intelligence quotient; HbA1c, glycated hemoglobin; T1D, type 1 diabetes

Correlation analysis revealed that AIQ was inversely related to age, diabetes duration, and HbA1c levels, and positively associated with parental education (both maternal and paternal) (Figure 4).

**Figure 5:**
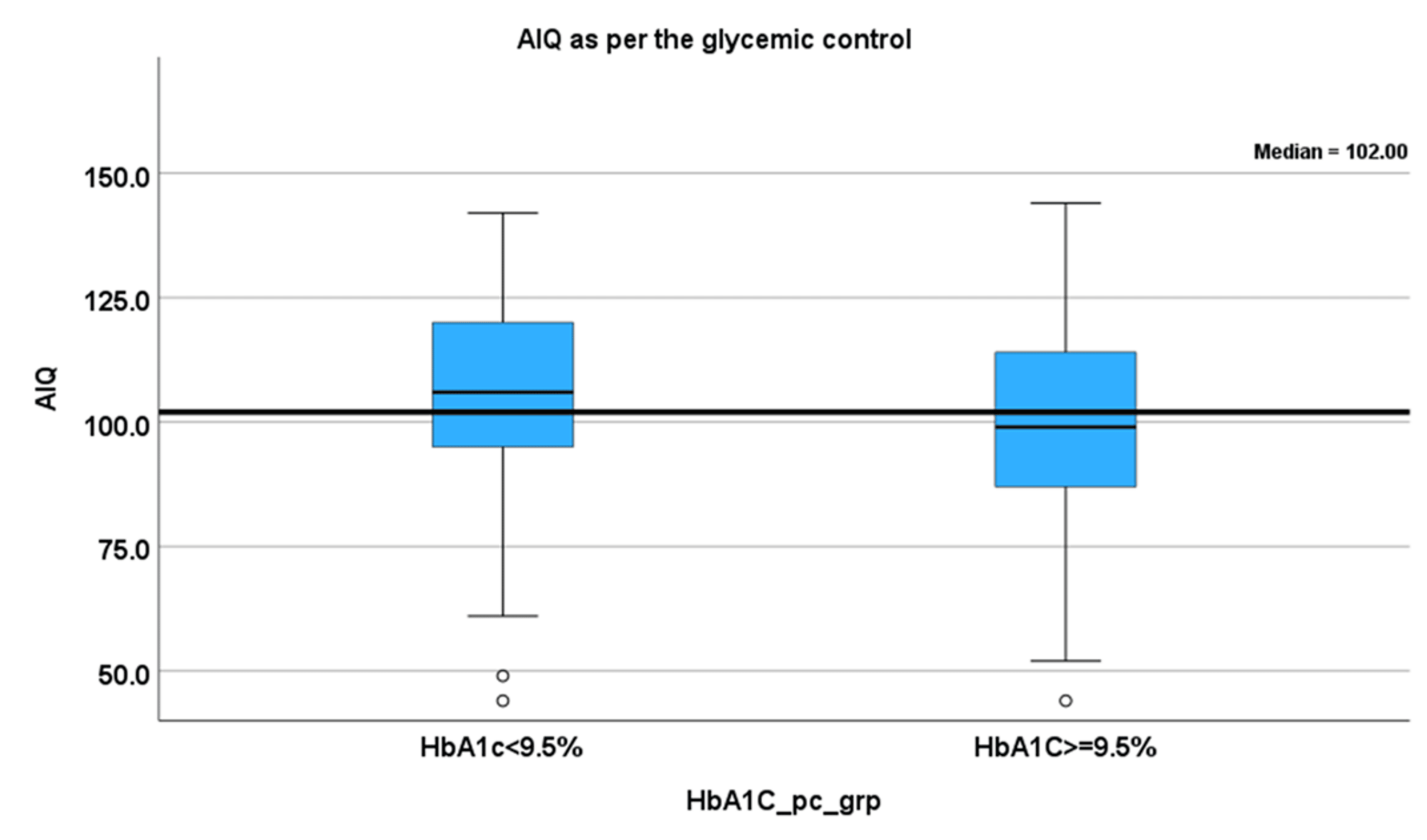
Median AIQ based on glycemic control categories Box plot comparing median AIQ across groups categorized by glycemic control levels (e.g., optimal vs. suboptimal based on ADA guidelines and according to the SEARCH for Diabetes in Youth Study) to assess cognitive differences associated with metabolic control. ADA, American Diabetes Association; AIQ, adjusted intelligence quotient; HbA1c, glycated hemoglobin

Participants with higher HbA1c levels exhibited lower AIQ scores compared to those with better glycemic control (Figure 5).

**Figure 6:**
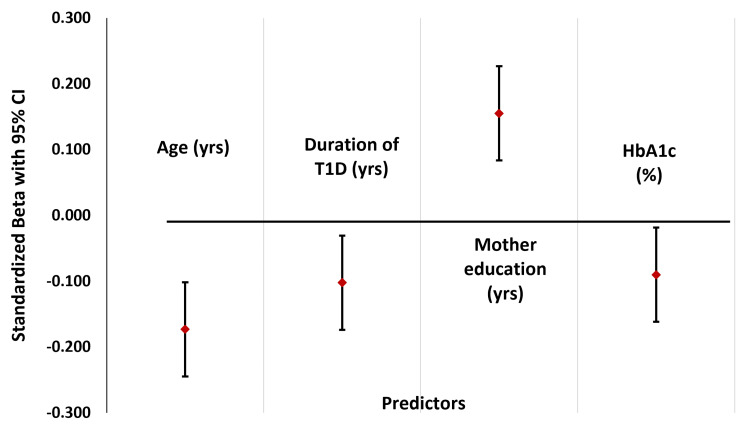
Predictors of AIQ in children with T1D Forest plot summarizing multivariable analysis results, indicating the independent predictors of AIQ among children and youth with T1D, including sociodemographic, clinical, and metabolic factors. AIQ, adjusted intelligence quotient; T1D, type 1 diabetes

Linear regression analysis further confirmed age, parental education, diabetes duration, and glycemic control (HbA1c) as significant predictors of AIQ in children and youth with T1D (Figure 6).

## Discussion

This study offers a comprehensive assessment of cognitive function, as measured by prorated IQ (AIQ), in a large cohort of Indian children and adolescents with T1D. While overall cognitive performance fell within the expected normative range, the analysis highlights meaningful associations between glycemic variables and cognitive outcomes. Specifically, older age, longer disease duration, and poor glycemic control were linked to lower AIQ, whereas higher parental education was associated with better cognitive scores. These findings emphasize the potential influence of both metabolic and socioenvironmental factors on cognitive development in youth with T1D.

Comparison with international studies

International research consistently highlights subtle cognitive differences in children with T1D, particularly in those with early onset and poor glycemic control. Longitudinal research conducted by Cato et al. in 2014 and 2016 examined young children with T1D and found that while overall cognitive scores generally fell within the normal range, chronic hypoglycemia and prior episodes of diabetic ketoacidosis (DKA) were linked to modest declines in executive function and verbal IQ. Over an 18-month follow-up, cognitive performance remained stable, but associations between poor glycemic control and reduced executive functioning became more evident [12,13]. These findings suggest that specific cognitive domains, especially those involving planning, organization, and verbal reasoning, may be more vulnerable to long-term metabolic imbalances.

Our observed average AIQ score supports the global trend of generally average overall cognitive functioning in children with T1D. However, consistent with international findings, our data suggest that children with persistently high HbA1c and longer disease duration tend to show lower cognitive performance, particularly in executive functioning. This reinforces the cognitive risk of sustained hypoglycemia, even in the absence of acute events such as DKA.

Schoenle et al. (2002) provide further longitudinal evidence that early diagnosis and poor metabolic control are critical risk factors [14]. Using the German version of the Hamburg Wechsler Intelligence Scale for Preschool Children, Children-Revised, as well as the Adaptives Intelligenz Diagnostikum, they found significant declines in verbal and performance IQ only in boys diagnosed with T1D before age 6. These declines were also associated with long-term poor glycemic control and high HbA1c levels. However, girls and children diagnosed after age six did not show similar cognitive deterioration. The limited sample size (38 girls and 26 boys) may have influenced these findings.

While our study emphasizes that prolonged exposure to high HbA1c and long disease duration are strong predictors of cognitive decline, no gender differences were noted. This underlines the need for early and consistent metabolic management to mitigate subtle but cumulative cognitive risks in children with T1D.

Parental education and IQ have also emerged as important correlates of child cognitive development across various populations. A study by Li et al. (2024) in children with autism spectrum disorder found that maternal education level significantly correlated with both IQ and adaptive behavior, whereas paternal education did not show a similar association [15]. Similarly, Sajewicz-Radtke et al. (2025) found that maternal education accounted for 18.23% of the variance in child IQ among a large mental health cohort, with child IQ increasing with age in children of better-educated parents [16]. These findings underscore the consistent role of maternal factors, particularly educational attainment, in shaping cognitive outcomes.

Despite these insights, there is a lack of both Indian and international studies investigating the relationship between parental education and child IQ in children with T1D. Our study is among the first to identify a link between maternal education and child IQ in a T1D cohort. This suggests that, beyond metabolic and disease-related factors, cognitive potential in children with T1D may also be influenced by environmental aspects of parental intelligence. Future research should explore this dimension further, ideally through longitudinal designs and larger, more diverse samples.

Comparison with Indian studies

Indian studies on cognitive function in children with T1D are limited but provide relevant benchmarks. Swaminathan et al. (2025) found significantly lower IQs, using Malin’s Intelligence Scale for Indian Children (MISIC), in children diagnosed before age 6, particularly those with poor glycemic control (HbA1c >9%), severe hypoglycemia, or recent DKA [17]. While our study also found that higher HbA1c was associated with lower AIQ, the overall mean AIQ remained within the normal range, suggesting that while glycemic extremes impact cognition, the effect may be attenuated in broader, more heterogeneous populations.

Also using MISIC, Puri et al. (2013) reported a mean IQ of 96.0 (SD 11.2) in 49 Indian children and adolescents with T1D, with lower scores linked to socioeconomic status [18]. Notably, while previous studies relied on MISIC, we employed the BKT, as literacy and verbal comprehension barriers in our cohort, especially among younger or non-school-going participants, posed challenges for language-heavy instruments.

Regarding the validity of our method, Patil et al. (2020) reported an IQ range of 80-129 in rural children aged 3-7 years, with no significant correlation between anthropometric indicators and IQ, despite high rates of malnutrition [9]. Their findings support the applicability of this method among younger cohorts in rural Indian settings and align with our observation that demographic and anthropometric variables did not significantly correlate with AIQ. By extending the age range to 4-22 years, our study builds on this foundation, offering a broader developmental perspective and enhancing the method’s utility in capturing cognitive trends across a wider span of growth and maturation.

Collectively, these Indian studies highlight that while biological factors such as glycemic control and diabetes duration are important, sociocultural elements, particularly parental education, consistently emerge as powerful determinants of cognitive outcomes.

Strengths and limitations

This study assessed IQ in a relatively large and diverse cohort (n = 413) of Indian children and youth with T1D, providing valuable insights into a population often underrepresented in cognitive research. The BKT, an Indian adaptation of the Stanford-Binet scale, was used for IQ assessment, ensuring greater cultural and linguistic relevance. The age range of our sample (4-22 years) aligns with the validated range for the BKT, compared with the narrower 6-15 years applicability of the more commonly used MISIC. The BKT’s lower dependence on literacy and schooling also made it more suitable for our heterogeneous sample, particularly participants from rural or lower socioeconomic backgrounds.

We integrated cognitive assessments with detailed sociodemographic, clinical, anthropometric, and lifestyle data, allowing for a multifactorial analysis of IQ determinants. The application of prorated/AIQ accounted for the Flynn effect, addressing concerns about the outdated norms of the BKT and enhancing the validity of the IQ scores. The use of both correlation and multivariate regression analyses enabled the identification of independent predictors of IQ, adjusting for potential confounders. By including participants from the “Sweetlings” program, which supports underprivileged children, the study ensured socioeconomic diversity, making the findings more generalizable within lower-resource Indian settings.

Despite adjustments, the BKT has not been updated since the 1960s. Even with prorated scoring, it may not fully capture contemporary cognitive challenges, particularly in a rapidly evolving educational context. The absence of a matched healthy control group limits direct comparison of IQ levels between children with and without T1D. The study’s cross-sectional design also limits causal inference; longitudinal studies would be better suited to track cognitive changes over time with disease progression or improved glycemic control.

Parental education and lifestyle factors (e.g., physical activity and sleep) were self-reported, potentially introducing recall or social desirability bias. While the BKT provides an overall IQ score and assesses several cognitive functions, it does not offer a full neuropsychological profile, including attention, executive function, or processing speed, which are often affected in T1D. As the study was conducted in a single tertiary care center in Western India, regional differences in education, healthcare access, and language may limit generalizability.

## Conclusions

This study highlights the complex relationship between chronic illness and cognitive development in children and adolescents with T1D. While IQ generally remained within the normative range, the findings suggest that sustained metabolic challenges, combined with socioenvironmental influences, may subtly shape cognitive functioning over time. These patterns reinforce the notion that T1D affects not only physical health but also neurodevelopmental trajectories, in ways that may not be immediately apparent in clinical settings. In resource-constrained environments, where access to tailored assessments and interventions is limited, these cognitive aspects may go unrecognized, contributing to long-term educational and psychosocial disparities.

To address this, routine cognitive screening and context-sensitive interventions should be considered part of comprehensive diabetes management. Supporting children not only through medical treatment but also by strengthening educational and family systems may help mitigate the cumulative cognitive load of living with a chronic condition. Investing in longitudinal research will be critical to understanding how cognitive outcomes evolve over time and to designing preventive strategies that are both culturally and developmentally appropriate. Ultimately, a more holistic approach to diabetes care can foster better long-term functioning and quality of life for young individuals navigating the dual challenges of illness and development.
